# Poisoning by Opium and Belladonna, Used as Injection

**Published:** 1830-10-01

**Authors:** 


### POISONING BY OPIUM AND BELLADONNA, USED AS AN INJECTION.

MADAME * * * *, æt. 22, had been tormented for some months by a dartrous eruption on the vulva. Having tried various means without relief, she applied a strong solution of opium and belladonna, which gave her some ease. On the 19th of November, 1829, she determined to use the narcotics in the form of enemata, and mixed a pint of the lotion with sufficient water to make three injections, each containing a scruple of opium and half an ounce of the leaves of belladonna. She took the first at eight o'clock in the evening, and the second and third shortly after the first; all three were returned, and some fæces were discharged. At half-past eight she went to bed, feeling rather confused. Her husband, a medical man, found her sleeping soundly at half-past nine, and did not disturb her. At midnight, however, finding her still buried in the deepest slumber, he became alarmed and sent for Dr. Solon. Her face was now extremely pale, the pupils dilated to the utmost, the tongue dry, deglutition difficult, the respirations short and frequent, the pulse small and 130. The limbs were perfectly motionless and the skin insensible to pinching or irritation of any kind. A purgative injection was immediately exhibited, a free bleeding practised from the arm, ten leeches placed behind each ear, sinapisms applied to the thighs and legs, and ether draughts administered by the mouth. At five o'clock next morning the patient opened her eyes, uttered a few unconnected words, and vomited some bilious matters. In the course of an hour she awoke, as from a painful dream, recollecting nothing of what had occurred. Vision was imperfect, she appeared to see things through a thick veil, and whenever she closed her eyes her ideas became confused and incoherent. We need not pursue the details, suffice it to say that the lady recovered from all her severe symptoms. The parts, however, to which the sinapisms had been applied, sloughed in consequence of the length of time they had been continued, and many months elapsed before she could walk about. The eruption on the vulva was quite cured by this severe discipline, and the patient subsequently went well through a confinement.

